# Subsurface detection of hair follicles in alopecia areata using optical coherence tomography

**DOI:** 10.1111/srt.13138

**Published:** 2022-01-12

**Authors:** Ai Ping Yow, Wellington Zhengdao Lee, Damon Wing Kee Wong, Hong Liang Tey

**Affiliations:** ^1^ Singapore Centre for Environmental Life Sciences Engineering Singapore Singapore; ^2^ National Skin Centre Singapore Singapore; ^3^ School of Chemical and Biomedical Engineering Nanyang Technological University Singapore Singapore; ^4^ Lee Kong Chian School of Medicine Nanyang Technological University Singapore Singapore; ^5^ Yong Loo Lin School of Medicine National University of Singapore Singapore Singapore

Dear Editors,

Alopecia areata (AA) is an autoimmune skin disorder that affects any hair‐bearing areas and results in localized nonscarring hair loss. It can occur in individuals at any age regardless of ethnicity with a lifetime risk of 1–2%.^1^ The loss of hair is caused by perifollicular inflammation, which is associated with cytokines and chemokines released by T cells.^2^, ^3^ Although spontaneous regrowth is possible in 80% of patchy AA, full resolution is slow.^4^ Patients with AA can be treated with various treatment options including a topical or intralesional steroid. While intralesional steroid injection is more effective and frequently used, it requires repeated injections every 4–6 weeks. In addition, patients may experience discomfort caused by the needle pricks during injection and the presence of steroid‐induced atrophy at the site of injection.

Optical coherence tomography (OCT) is a noninvasive imaging technique based on the principle of optical interferometry using low‐coherence light.^5^ This technique has been widely used in ophthalmology to enable the diagnosis and management of retinal diseases.^6^ OCT was then extended to dermatology to visualize cellular and morphological changes beneath the human skin.^7^, ^8^, ^9^, ^10^, ^11^, ^12^, ^13^ Further studies have also shown the application of OCT to visualize hair follicles at nonscalp areas,^4^, ^8^, ^14^ among which, Boone et al. had described the hair follicles as whorled, hollow structures with a central refractile hair shaft on enface OCT images.^8^ In cross‐sectional OCT images, however, hair follicles appear as dark regions within the bright‐appearing dermis.^15^ Recently, Garcia Bartels et al. demonstrated the use of OCT to analyze structural abnormalities of hair shafts on patients with AA.^4^ However, there have not been studies on in vivo OCT analysis of hair follicles below the scalp surface.

As such, we aim to investigate the use of OCT to characterize features of subsurface hair follicles at the dermoepidermal junction (DEJ) for better management and treatment of AA. We first identify the features of subsurface hair follicles at the DEJ with OCT and evaluate the usability of OCT in detecting subsurface hairs in AA patches.

A retrospective cross‐sectional study was conducted at the National Skin Centre, Singapore and approved by the National Healthcare Group's Domain Specific Ethics Review Board (reference 2015/00738). It comprised 20 patients (12 male, 8 female) aged 9–68 years (median age of 37), with the ethnicity percentages reflecting the racial distribution in Singapore (Chinese 75%, Malay 15%, Indian 10%). Most patients had one or more treatments before performing the OCT scan, and the majority (80%) had intralesional triamcinolone injections. Other therapies include topical steroids (betamethasone valerate 0.1% scalp lotion, clobetasol scalp application 0.05%), minoxidil 2% or 5% lotion, oral prednisolone, and immunotherapy with diphenylcyclopropenone.

We performed OCT scans on the patients using a high‐definition OCT (Skintell, Agfa Healthcare, Belgium) with an enhanced resolution of 3 μm.^8^ Compared to reflectance confocal microscopy, high‐definition OCT enables three‐dimensional (3D) visualization of skin tissue up to a depth of 570 μm where the upper dermis is located.^7^, ^8^ OCT scans with excessive shadow artifacts due to the presence of hairs above the scalp and motion artifacts were excluded. In addition, scans with noticeable misalignments of the OCT imaging probe were also excluded. A 3D segmentation algorithm, which was developed previously,^16^ was applied to separate the epidermis and the dermis for each high‐definition OCT scan. The segmented epidermis and dermis were then vertically projected into two‐dimensional (2D) en face images of scalp surface and DEJ, respectively. From the generated en face images, we first characterized the appearance of hair follicular structures on the scalp surface. Next, using ImageJ software (National Institutes of Health, USA), we performed manual matching of the identified structures from the scalp surface to the DEJ. Finally, we tabulated the number of hair follicles identified on both the scalp surface and DEJ.

A total of 30 OCT scans from individual AA patches of the 20 patients were analyzed, and OCT features of the hair and hair follicles at various depths were observed. We observed that the scalp appeared as a bright surface and the hair follicles were seen as dark and hollow lumens (Figure 1A). The hair follicles can be either circular or elliptical in shape. It was also observed that inside the dark lumens, there were bright and reflective rings, which represented the outer sheath and cortex of hair shafts. At DEJ, the amorphous nature of collagenaceous ground substance could be appreciated, and the outer sheath of hair shafts were appeared to be less reflective (Figure 1B).

**FIGURE 1:**
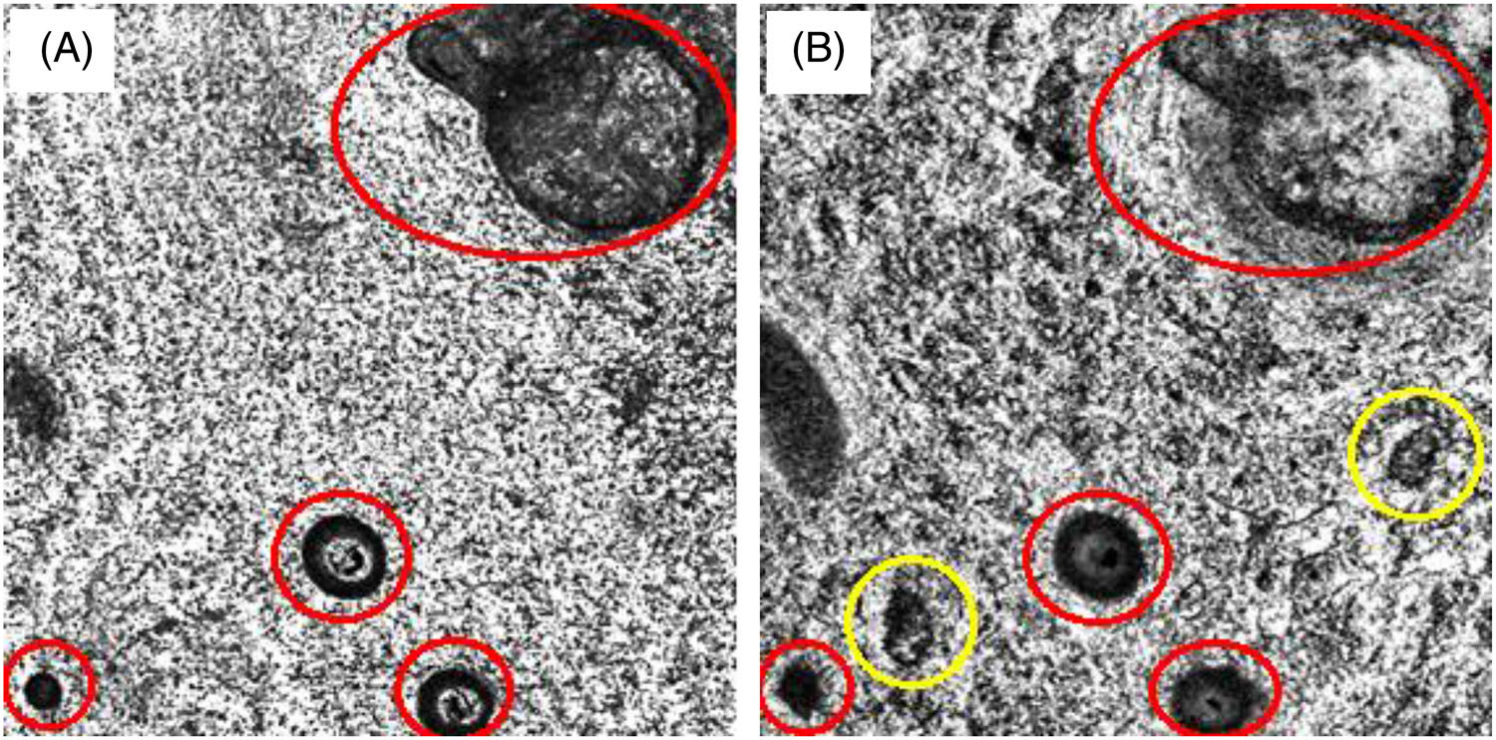
En face image of an alopecia areata (AA) patch using 3D segmentation. (A) Scalp surface with hair follicles and hair (circled red). (B) Dermoepidermal junction (DEJ) with hair follicular structures (circled yellow), which are not visible on the scalp surface

From the 2D enface image of DEJ, we found similar hair follicular structures, which matched the exact sites of hair follicles identified at the scalp surface. We also identified additional hair follicles at the DEJ that were not visible at the scalp surface. Of the 30 OCT scans analyzed, 22 had more subsurface hair follicles at the DEJ compared to the scalp surface (Table 1), and these subsurface structures could be an indication of growing hair follicles in AA. Further prospective studies can help to correlate OCT scans with subsequent clinical improvement of AA. OCT may potentially be used in clinical settings to monitor treatment response and provide reassurance to patients who have responded. Treatments such as intralesional steroid injections can be stopped earlier to avoid further side effects and reduce the burden of treatment. This process can also be applied to other types of alopecia disorders and to monitor therapeutic response especially when using drugs with potentially severe side effects.

**TABLE 1:** Twenty‐two (highlighted in yellow) of 30 optical coherence tomography (OCT) scans had more subsurface hair follicles at the dermoepidermal junction (DEJ) that were not detected on the scalp surface

**Number of hair follicles detected on scalp surface and dermoepidermal junction (DEJ)**					
Image volume	Scalp surface	DEJ	Image volume	Scalp surface	DEJ
1	3	8	16	4	7
2	2	4	17	2	2
3	4	7	18	2	2
4	1	1	19	1	1
5	2	4	20	1	2
6	3	5	21	1	3
7	2	3	22	3	3
8	2	6	23	2	4
9	2	6	24	3	3
10	1	1	25	3	4
11	2	2	26	1	3
12	3	5	27	4	8
13	1	2	28	3	5
14	2	6	29	3	6
15	3	6	30	6	9

We also observed that some subsurface hair follicles at the DEJ appeared to be hollow at the center. It is likely that new hair follicles with hairs were still growing beneath the DEJ. Larger hair follicular structures were most likely terminal hairs, while the smaller structures were likely vellus hairs occurring in AA. Further analysis was not performed on the dermal layer as the surrounding dermal ground substance posed a challenge for distinguishing the hair follicles.

In conclusion, OCT enables visualization of hair follicles at the DEJ and detection of subsurface hair follicular structures, which are not visible on the scalp surface. The latter likely reflects the regrowing of hair follicles in AA. Further studies can help correlate this finding with clinical improvement of AA, and OCT may also be used to monitor treatment response.

## CONFLICT OF INTEREST

All authors have no conflict of interests or financial disclosures to declare.
